# Supplementary material for: Proteogenomics of different urothelial bladder cancer stages reveals distinct molecular features for papillary cancer and carcinoma in situ
Source: Nat Commun. 2023 Sep 13;14:5670. doi: 10.1038/s41467-023-41139-3 (PMC10499981; doi:10.1038/s41467-023-41139-3)
Supplement: Supplementary file 1 — Supplementary Information [file 41467_2023_41139_MOESM1_ESM.pdf]

Supplementary Figure 1

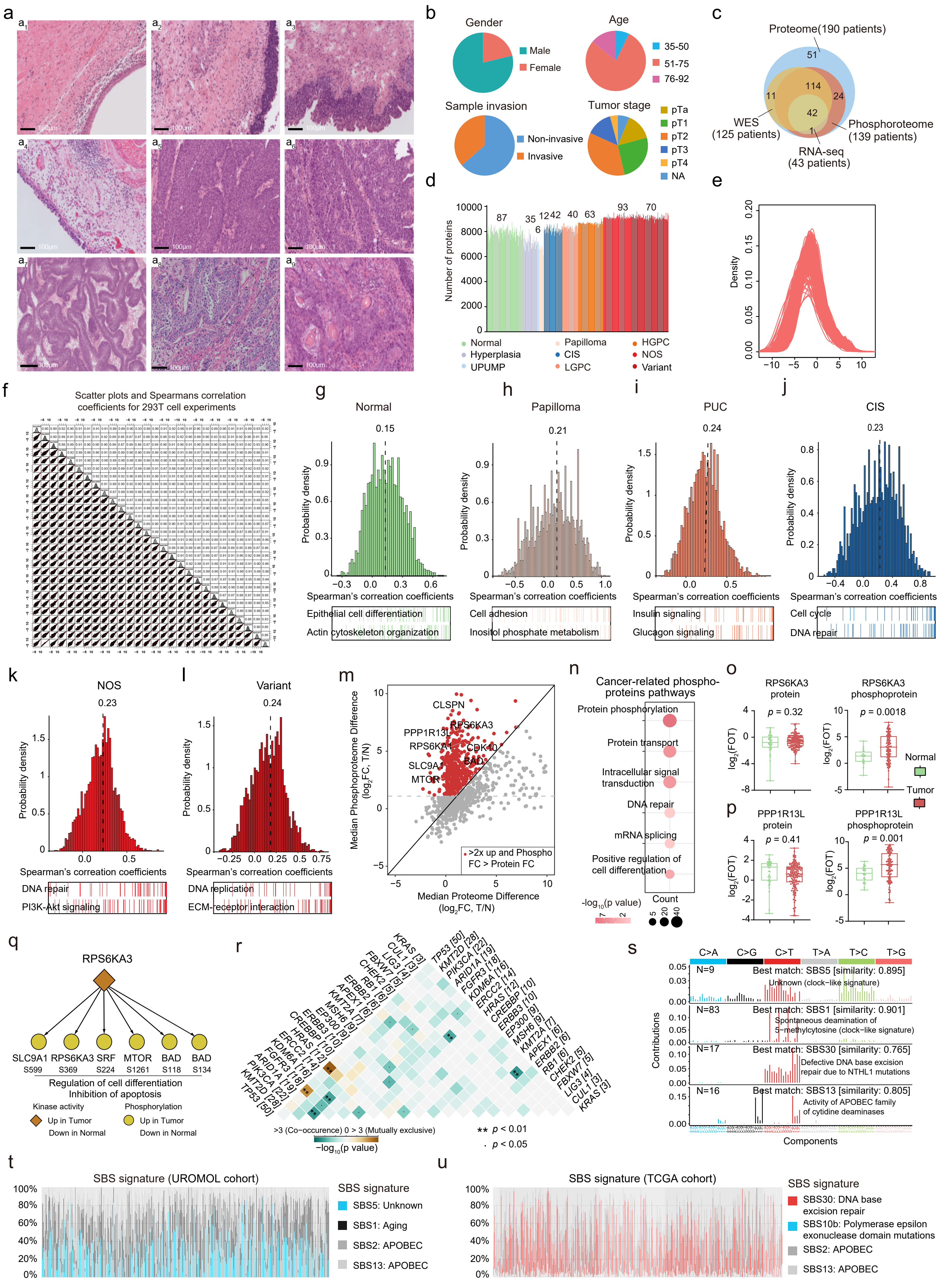

Supplementary Figure 1. Proteogenomic landscape of early urothelial bladder cancer cohort. Related to Figure 1.

(A) H&E-stained slides of different urothelial bladder cancer (UC) tissues (analyzed patients: n = 9). The scale bar is 100 µm. (a1) An example of morphologically normal human urothelium (Normal). (a2) An example of urothelial hyperplasia (Hyperplasia). (a3) An example of urothelial proliferation of uncertain malignant potential (UPUMP). (a4) An example of carcinoma *in situ* (CIS). (a5) An example of non-invasive low-grade papillary cancer (LGPC). (a6) An example of non-invasive high-grade papillary cancer (HGPC). (a7) An example of inverted urothelial papilloma (Papilloma). (a8) An example of invasive cancer without otherwise specified histology (NOS). (a9) An example of invasive cancer with variant histology (Variant). **(B)** Age, gender, tumor stage, and sample invasion status proportions in the cohort. **(C)** Venn diagram summary of the number of patients used in proteomics, phosphoproteomics, whole-exome sequencing, and RNA-Seq experiments. **(D)** The identified protein numbers of 448 samples. **(E)** Distribution of protein abundances in 448 samples by density plot. A unimodal distribution (dip test) was observed. All samples passed proteomic quality control. **(F)** Quality control of mass spectrometry using tryptic digest of 293T cells. The top-left half of the panel represents the pairwise Spearman's correlation coefficients of samples and the bottom-right half of the panel depicts the pairwise scatter plots from sample comparison. **(G-L)** Top panel: phosphoprotein-protein correlation in Normal (G), Papilloma (H), PUC (I), CIS (J), NOS (K), and Variant (L). Bottom panel: pathways in which positively correlated proteins were involved in Normal (G), Papilloma (H), PUC (I), CIS (J), NOS (K), and Variant (L). **(M)** Fold-changes of proteins and phosphoproteins, and their correlations in tumor (T) and normal (N). Red dots: phosphoproteins are greater than 2-fold changes in tumor compared to normal, and changes of phosphoproteins abundance are greater than changes of their corresponding protein abundance. **(N)** Pathways enriched with the phosphoproteins (red dots). (**O**) Boxplots showing the expression of RPS6KA3 protein (left, n = 177) and RPS6KA3 phosphoprotein (right, n =122, p = 1.8E-3) in tumor and normal samples (two-sided Wilcoxon rank-sum test). Boxplots show median (central line), upper and lower quartiles (box limits), 1.5× interquartile range (whiskers). (**P**) Boxplots showing the expression of PPP1R13L protein (left, n = 174) and PPP1R13L phosphoprotein (right, n = 132, p = 0.001) in tumor and normal samples (two-sided Wilcoxon rank-sum test). Boxplots show median (central line), upper and lower quartiles (box limits), 1.5× interquartile range (whiskers). (**Q**) Diagram illustrating

differences between tumor and normal in terms of phosphorylation abundance and kinase activity for RPS6KA3. **(R)** The co-mutations and mutually exclusive mutations (two-sided Fisher's exact test, p = 0.0032 (*TP53* and *HRAS*), p = 0.0017 (*TP53* and *FGFR3*)) **(S)** Mutational spectrum of the four mutational signatures extracted by Sigminer analysis in our cohort. **(T)** The relative percentage of each mutational signature profile of patients in UROMOL cohort. **(U)** The relative percentage of each mutational signature profile of patients in TCGA cohort. Source data are provided as a Source Data file.

Supplementary Figure 2

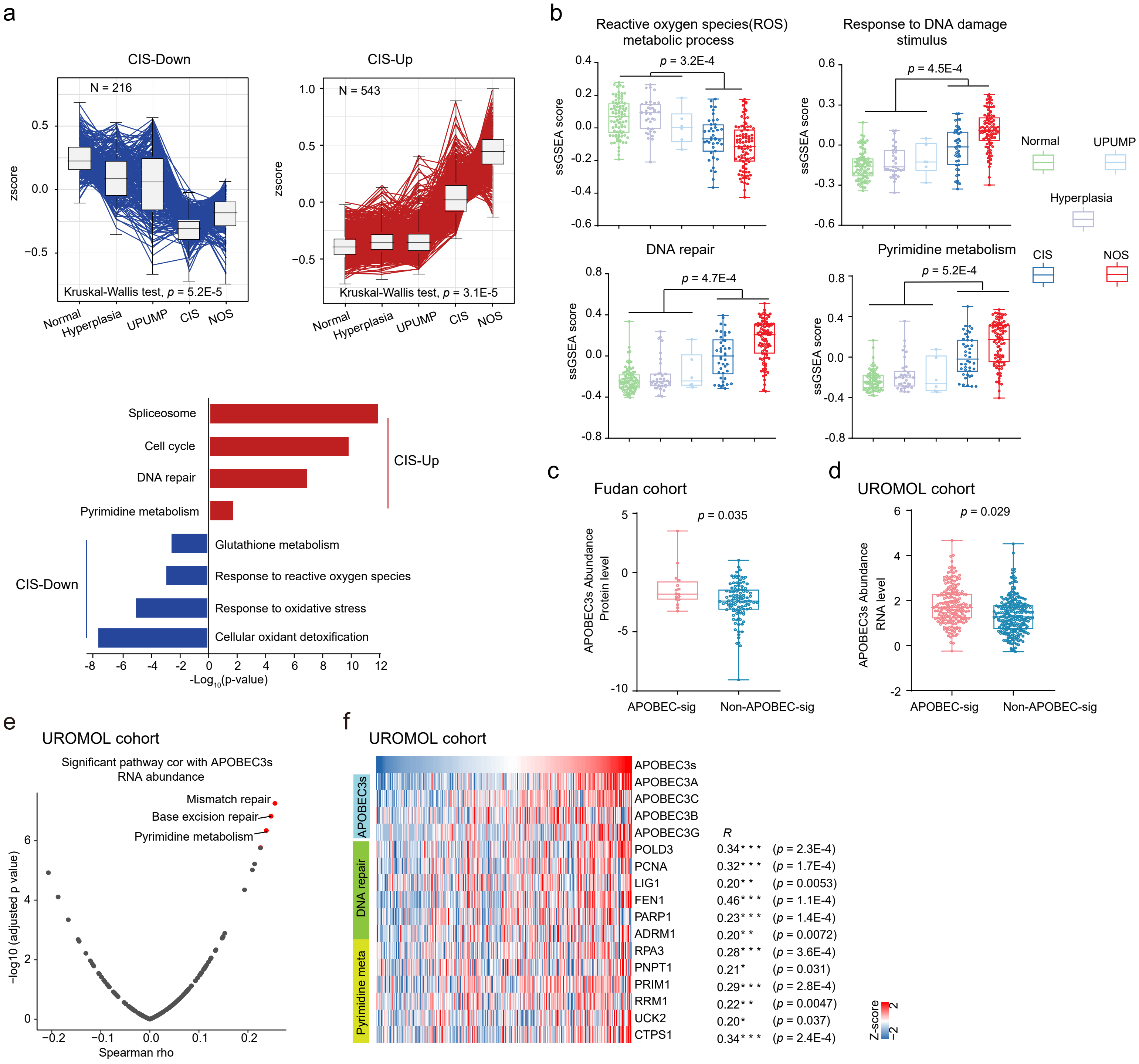

Supplementary Figure 2. DNA damage signaling related to APOBEC signature was a key signaling pathway in the progression of CIS. Related to Figure 2.

(A) Left: Line plots and boxplots of selected gene sets with up- or downregulation trend during CIS progression at protein level (Kruskal-Wallis test). Right: Pathways enriched for up- or downregulation trend proteins during CIS progression. Boxplots show median (central line), upper and lower quartiles (box limits), 1.5× interquartile range (whiskers). **(B)** Boxplots illustrating the significantly up- or downregulation pathways in Normal (green, n = 87), Hyperplasia (purple, n = 35), UPUMP (blue, n = 6), CIS (navy, n = 42), and NOS (red, n = 93) (two-sided Wilcoxon rank-sum test, p = 3.2E-4 (ROS metabolic process), p = 4.5E-4 (response to DNA damage stimulus), p = 4.7E-4 (DNA repair), p = 5.2E-4 (pyrimidine metabolism)). Boxplots show median (central line), upper and lower quartiles (box limits), 1.5× interquartile range (whiskers). **(C)** Expression profiles of APOBEC3s in APOBEC-signature-containing samples (n = 16) and Non-APOBEC-signature-containing samples (n = 109) in our cohort (two-sided Wilcoxon rank-sum test, p = 0.035). Boxplots show median (central line), upper and lower quartiles (box limits), 1.5× interquartile range (whiskers). **(D)** Expression profiles of APOBEC3s in APOBEC-signature-containing samples and Non-APOBEC-signature-containing samples in UROMOL cohort (two-sided Wilcoxon rank-sum test, p = 0.029). Boxplots show median (central line), upper and lower quartiles (box limits), 1.5× interquartile range (whiskers). **(E)** Volcano plot showing the correlation between enriched KEGG pathways ssGSEA scores and APOBEC3s abundance (two-sided Spearman's correlation test) in UROMOL cohort. **(F)** Heatmap showing relative abundance and Spearman's correlation between APOBEC3s and DNA repair and pyrimidine metabolism molecules in UROMOL cohort. At the right was the Spearman's correlation between APOBEC3s and the proteins involved in DNA repair and pyrimidine metabolism (two-sided Spearman's correlation test). *$p < 0.05$ is considered statistically significant. *$p < 0.05$, **$p < 0.01$, ***$p < 0.001$, ****$p < 0.0001$, ns > 0.05. Source data are provided as a Source Data file.

Supplementary Figure 3

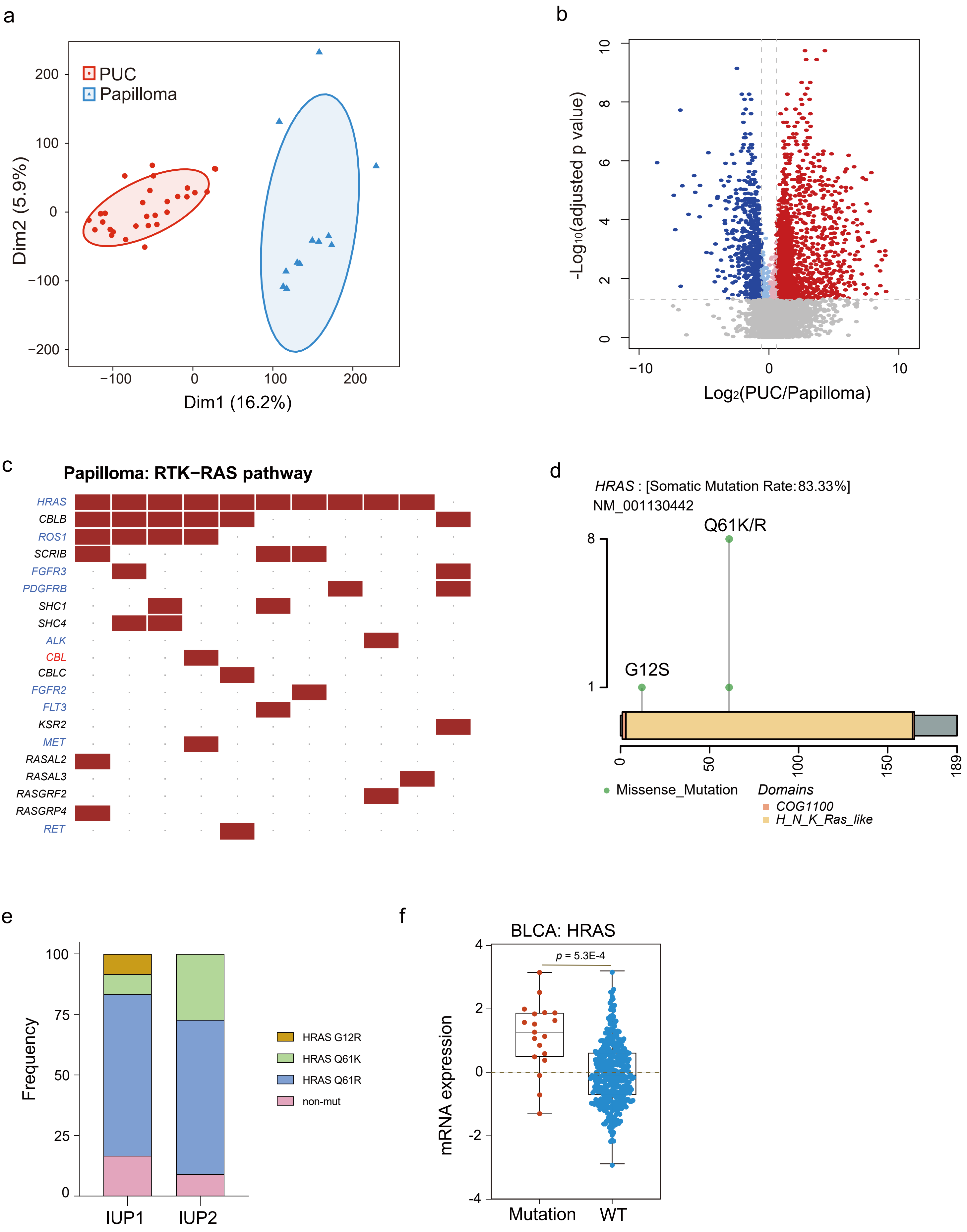

Supplementary Figure 3. Proteogenomic Profiles Distinguished Papilloma from papillary urothelial cancer (PUC). Related to Figure 3.

(A) PCA analysis of papilloma and papillary urothelial cancer (PUC). **(B)** Volcano plot showing the differential proteins of papilloma and PUC (two-sided Wilcoxon rank-sum test, Benjamini-Hochberg (BH)-adjusted $p < 0.05$). (**C**) The genes of RTK-RAS pathway mutated in papilloma tumors. **(D)** Mutational hotspots of *HRAS* in our cohort. **(E)** Bar plot showing the different mutational hotspot of *HRAS* mutation between our cohort (IUP1) and New York cohort (IUP2). **(F)** Box plot showing the mRNA abundance of HRAS in patients with *HRAS* mutations (n = 19) and without *HRAS* mutations (n = 389) in TCGA cohort (two-sided Wilcoxon rank-sum test, p = 5.3E-4). Boxplots show median (central line), upper and lower quartiles (box limits), 1.5× interquartile range (whiskers). Source data are provided as a Source Data file.

Supplementary Figure 4

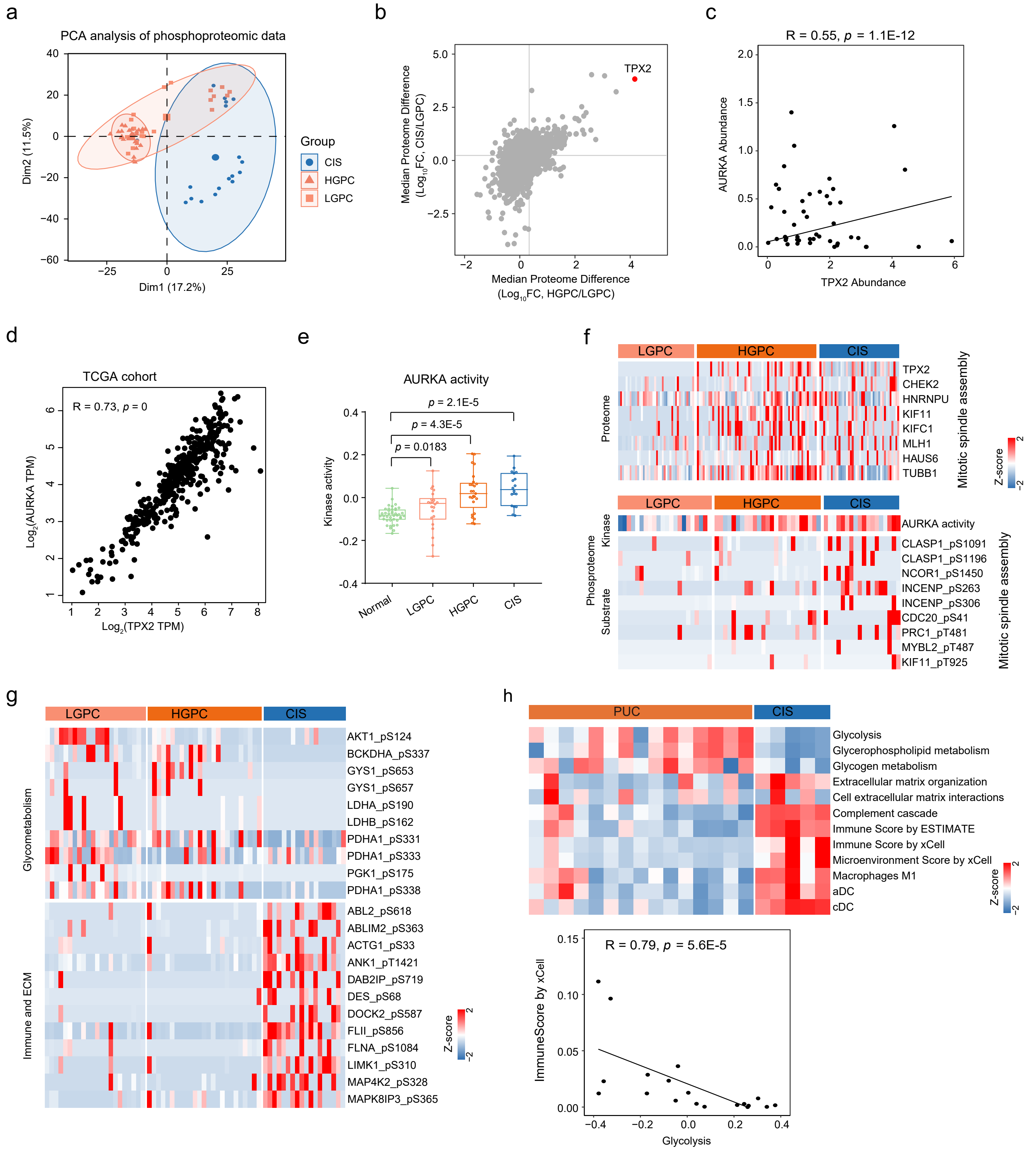

Supplementary Figure 4. The different metabolic and immune characteristics of papillary urothelial cancer and CIS. Related to Figure 4.

(A) PCA analysis of phosphoproteomics (2,222 phosphoproteins) among LGPC (n = 22), HGPC (n = 25), and CIS (n = 18). **(B)** Fold-changes of proteins in LGPC, HGPC, and CIS (two-sided Wilcoxon rank-sum test). **(C)** Correlation of AURKA protein abundance with TPX2 protein abundance in our cohort (two-sided Spearman's correlation test). **(D)** Correlation of AURKA mRNA abundance with TPX2 mRNA abundance in TCGA BLCA cohort (two-sided Spearman's correlation test). (**E**) The kinase activity of AURKA in Normal (n = 41), LGPC (n = 22), HGPC (n = 25), and CIS (n = 18) groups. (two-sided Wilcoxon rank-sum test). Boxplots show median (central line), upper and lower quartiles (box limits), 1.5× interquartile range (whiskers). (**F**) Heatmap of the protein abundance (top panels) and phosphosites abundance (bottom panels) of mitotic spindle assembly related genes. (**G**) Heatmap of the phosphosites abundance related to glycometabolism proteins (top panels) and immune and extracellular matrix proteins (bottom panels) in LGPC, HGPC, and CIS tissues. (**H**) Heatmap illustrating ssGSEA scores of selected pathways differentially expressed between PUC and CIS in Dyrskjøt's cohort (two-sided Wilcoxon rank-sum test). Source data are provided as a Source Data file.

Supplementary Figure 5

a

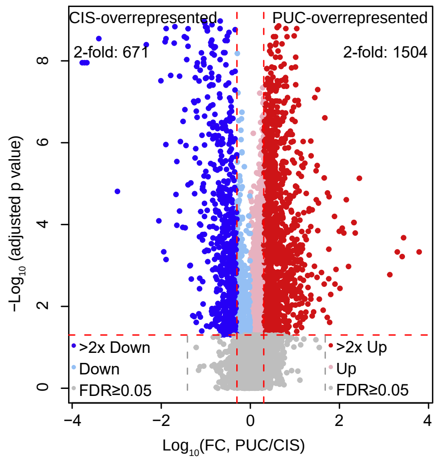

c

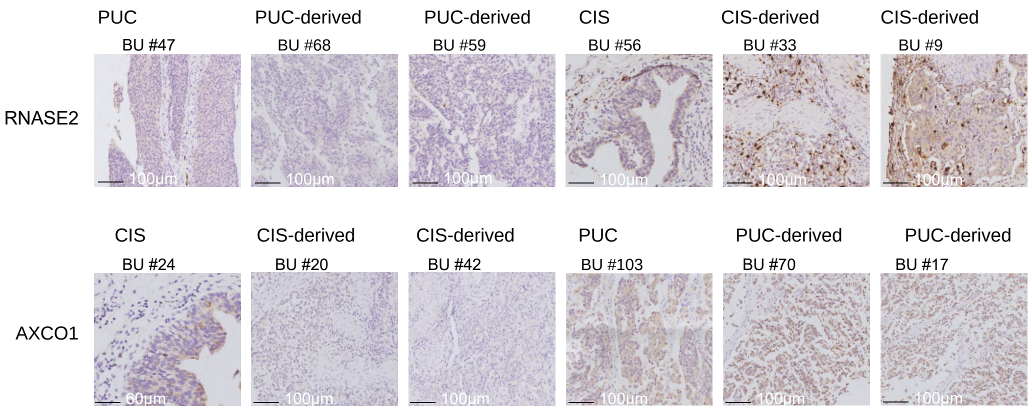

b

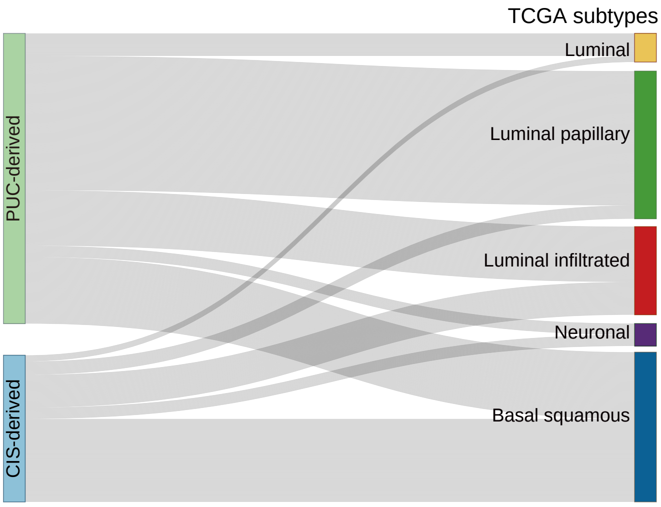

Supplementary Figure 5. The distinction of PUC - and CIS - derived tumors and their association with clinical outcomes. Related to Figure 5.

(A) Proteins abundance differences between PUC or CIS (two-sided Wilcoxon rank-sum test, BH-adjusted $p < 0.05$). **(B)** Sankey diagram analysis of PUC-derived/CIS-derived subtypes and TCGA subtypes. **(C)** IHC profiling of the classifier model proteins in UC. FFPE sections were stained for RNASE2 and ACOX1 protein markers in UC tumor tissues (analyzed patients: n = 6). The scale bar indicates 100 μm. Source data are provided as a Source Data file.

Supplementary Figure 6

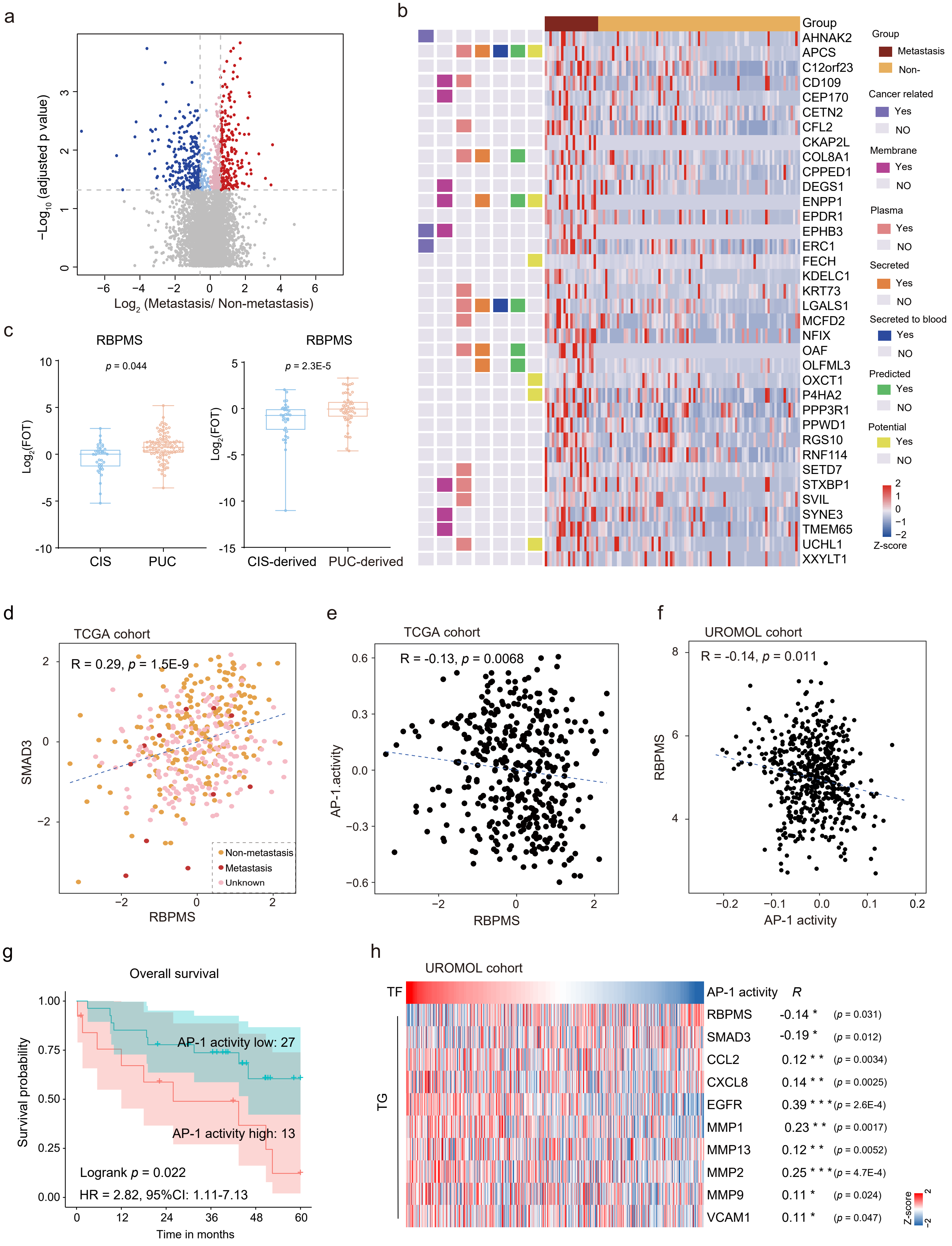

Supplementary Figure 6. Loss of RBPMS potentially driving tumor metastasis. Related to Figure 6. **(A)** Volcano plot showing the differential proteins of metastatic and non-metastatic tumors (two-sided Wilcoxon rank-sum test, BH-adjusted $p < 0.05$). **(B)** Heatmap of proteins significantly upregulated in metastasis tumors. At the left was the annotation from Human Proteome Atlas (HPA). **(C)** Boxplots showing the expression of RBPMS protein in CIS (n = 37) and PUC samples (n = 102) (left, $p = 0.044$) or CIS-derived (n = 33) and PUC-derived (n = 53) samples (right, $p = 2.3E-5$) (two-sided Wilcoxon rank-sum tests). Boxplots show median (central line), upper and lower quartiles (box limits), 1.5× interquartile range (whiskers). **(D)** Correlation of RBPMS mRNA abundance with SMAD3 mRNA abundance in TCGA cohort (two-sided Spearman's correlation test). **(E)** Correlation of RBPMS mRNA abundance with AP-1 activity in TCGA cohort (two-sided Spearman's correlation test). **(F)** Correlation of RBPMS mRNA abundance with AP-1 activity in UROMOL cohort (two-sided Spearman's correlation test). **(G)** Overall survival analysis of high AP-1 activity (n = 13) group versus low AP-1 activity (n = 27) group (two-sided log-rank test). 95% confidence interval (CI) and hazard ratios (HR) were also presented. **(H)** Heatmap showing the estimated AP-1 activity and the mRNA abundance of the target genes of AP-1 in UROMOL cohort. At the right was the Spearman's correlation between AP-1 activity and the target genes of AP-1 (two-sided Spearman's correlation test). $*p < 0.05$ is considered statistically significant. $*p < 0.05$, $**p < 0.01$, $***p < 0.001$, $****p < 0.0001$, ns > 0.05. Source data are provided as a Source Data file.

Supplementary Figure 7

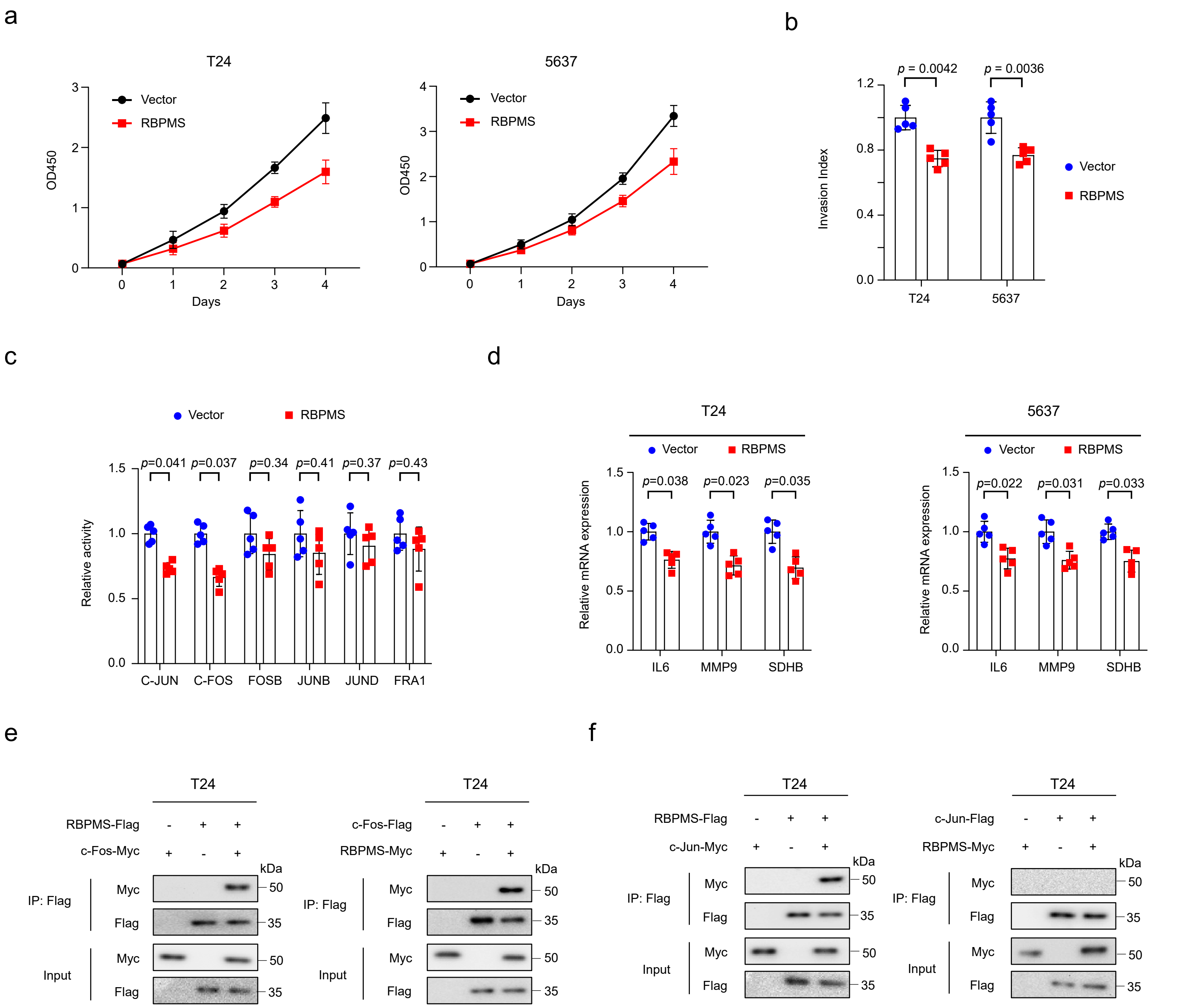

Supplementary Figure 7. The deficiency of RBPMS promoted UC progress through activating AP-1 transcription factors. Related to Figure 7.

(A) Cell Counting Kit-8 assays were performed to investigate the proliferation ability of T24 and 5637 cells after RBPMS overexpression treatment, measuring the OD450 value (n=5 repeats per group, two-sided Student's t-test, mean ± SEM). **(B)** The invasion ability of T24 and 5637 cells were detected after RBPMS overexpression treatment, measuring the invasion ability (n=5 repeats per group, two-sided Student's t-test, mean ± SEM). **(C)** After RBPMS overexpression treatment, the intracellular activities of AP-1 family members were measured by semi-quantitative colorimetric kit (n=5 repeats per group, two-sided Student's t-test, mean ± SEM). **(D)** After RBPMS overexpression treatment, the expression of IL-6, MMP9 and SDHB in T24 and 5637 cells were analyzed by RT-qPCR to indicate mRNA levels (n=5 repeats per group, two-sided Student's t-test, mean ± SEM). **(E)** T24 cells were cotransfected with FLAG-tagged RBPMS, Myc-tagged c-Fos, FLAG-tagged c-Fos, Myc-tagged RBPMS. Co-IP was performed using anti-FLAG and anti-Myc, followed by immunoblot with the indicated antibodies. **(F)** T24 cells were cotransfected with FLAG-tagged RBPMS, Myc-tagged RBPMS, Myc-tagged c-Jun and FLAG-tagged c-Jun. Co-IP was performed using anti-FLAG and anti-Myc, followed by immunoblot with the indicated antibodies. *$p < 0.05$ is considered statistically significant. *$p < 0.05$, **$p < 0.01$, ***$p < 0.001$, ****$p < 0.0001$, ns > 0.05. Source data are provided as a Source Data file.
