# Supplementary material for: Proteogenomics of different urothelial bladder cancer stages reveals distinct molecular features for papillary cancer and carcinoma in situ
Source: Nat Commun. 2023 Sep 13;14:5670. doi: 10.1038/s41467-023-41139-3 (PMC10499981; doi:10.1038/s41467-023-41139-3)
Supplement: Supplementary file 3 — Description of Additional Supplementary Files [file 41467_2023_41139_MOESM3_ESM.pdf]

Description of Supplementary Datasets

Supplementary Data Legends

File Name: Supplemental data 1

Description: Clinicopathologic information and multi-omics data of UC patients.

(A) Clinical data. **(B)** The sheet contained information of mutations identified by WES in the 125 UC patients. **(C)** Genome signature analysis. **(D)** Proteome quantification matrix. **(E)** Phosphoproteome quantification matrix. **(F)** Transcriptome quantification matrix.

File Name: Supplemental data 2

Description: The variation of DNA damage response signaling and APOBEC mutational signature during the CIS progression.

(A) List of proteins used for principal component analysis. **(B)** List of DNA damage response (DDR) marker phosphoproteins and their relative intensities. **(C)** List of APOBEC3s proteins and their relative intensities in APOBEC-sig and Non-APOBEC-sig samples. **(D)** List of APOBEC3s relative intensities and DDR score in different sample types.

File Name: Supplemental data 3

Description: Differentially expressed proteins, and phosphoproteins between papilloma and papillary urothelial cancer (PUC).

(A) List of proteins used for principal component analysis. **(B)** List of differentially expressed proteins (two-sided Wilcoxon rank-sum test, Benjamini-Hochberg (BH)-adjusted $p < 0.05$) in papilloma and PUC. **(C)** List of differentially expressed phosphoproteins (two-sided Wilcoxon rank-sum test, BH-adjusted $p < 0.05$) in papilloma and PUC. **(D)** List of differentially expressed pathways in papilloma and PUC.

File Name: Supplemental data 4

Description: Differentially expressed proteins among LGPC, HGPC and CIS samples.

(A) List of proteins used for principal component analysis. **(B)** List of differentially expressed proteins (transcription factors) (two-sided Wilcoxon rank-sum test, BH-adjusted $p < 0.05$) in PUC and CIS. **(C)**

Data analysis by xCell. **(D)** Sample-specific gene set enrichment analysis pathway scores of selected pathways differentially expressed among LGPC, HGPC, and CIS.

File Name: Supplemental data 5

Description: List of signatures of the classifier model for distinguishing invasive tumors as PUC-derived and CIS-derived tumors.

(A) List of differentially expressed proteins (two-sided Wilcoxon rank-sum test, BH-adjusted $p < 0.05$) in PUC and CIS. **(B)** List of signatures of the classifier model in the discovery cohort. **(C)** List of the classifier model genes that classified the origin of invasive tumors as CIS-derived and PUC-derived in the TCGA cohort.

File Name: Supplemental data 6

Description: Loss of RBPMS potentially driving tumor metastasis.

(A) List of differentially expressed proteins (two-sided Wilcoxon rank-sum test, BH-adjusted $p < 0.05$) in non-metastasis tumors and metastasis tumors. **(B)** Samples with 8p12 deletion. **(C)** List of the protein abundance of 9 cis-effects in 8p12. **(D)** List of the differentially expressed proteins of 9 cis-effects in metastatic and non-metastatic tumors (two-sided Wilcoxon rank-sum test, BH-adjusted $p < 0.05$).
