# Supplementary material for: Proteogenomics of different urothelial bladder cancer stages reveals distinct molecular features for papillary cancer and carcinoma in situ
Source: Nat Commun. 2023 Sep 13;14:5670. doi: 10.1038/s41467-023-41139-3 (PMC10499981; doi:10.1038/s41467-023-41139-3)
Supplement: Supplementary file 10 — Reporting Summary [file 41467_2023_41139_MOESM10_ESM.pdf]

nature portfolio

Corresponding author(s): Chen Ding
Yingyong Hou
Jianyuan Zhao
Jianming Guo
Jun Hou

Last updated by author(s): Jul 31, 2023

Reporting Summary

Nature Portfolio wishes to improve the reproducibility of the work that we publish. This form provides structure for consistency and transparency in reporting. For further information on Nature Portfolio policies, see our Editorial Policies and the Editorial Policy Checklist.

Statistics

For all statistical analyses, confirm that the following items are present in the figure legend, table legend, main text, or Methods section.

n/a	Confirmed	
☐	☒	The exact sample size (*n*) for each experimental group/condition, given as a discrete number and unit of measurement
☐	☒	A statement on whether measurements were taken from distinct samples or whether the same sample was measured repeatedly
☐	☒	The statistical test(s) used AND whether they are one- or two-sided<br>*Only common tests should be described solely by name; describe more complex techniques in the Methods section.*
☒	☐	A description of all covariates tested
☐	☒	A description of any assumptions or corrections, such as tests of normality and adjustment for multiple comparisons
☐	☒	A full description of the statistical parameters including central tendency (e.g. means) or other basic estimates (e.g. regression coefficient) AND variation (e.g. standard deviation) or associated estimates of uncertainty (e.g. confidence intervals)
☐	☒	For null hypothesis testing, the test statistic (e.g. *F*, *t*, *r*) with confidence intervals, effect sizes, degrees of freedom and *P* value noted<br>*Give P values as exact values whenever suitable.*
☒	☐	For Bayesian analysis, information on the choice of priors and Markov chain Monte Carlo settings
☐	☒	For hierarchical and complex designs, identification of the appropriate level for tests and full reporting of outcomes
☐	☒	Estimates of effect sizes (e.g. Cohen's *d*, Pearson's *r*), indicating how they were calculated

Our web collection on statistics for biologists contains articles on many of the points above.

Software and code

Policy information about availability of computer code

Data collection	The proteomic raw data were performed by Q Exactive HF-X Mass Spectrometer, Thermo Fisher Scientific. The phosphoproteomic raw data were performed by Q Exactive HF-X Mass Spectrometer, Thermo Fisher Scientific. The whole exon sequencing data were obtained through, Illumina Novaseq 6000. The RNA-seq data were obtained through, Illumina Novaseq 6000.
Data analysis	The data analysis was performed by programming language R (version 3.5.1). Most of them used broadly applied R packages and others used self-made R scripts according published papers: survminer (v0.2.4) for survival analysis, Hmisc (v4.5-0) for spearman's correlation calculating, ggplot2 (v3.3.5) for scatter plot. Gene annotation was performed using online tools DAVID 6.8. Results from the Fast Large Margin classifier model were built and validated using RapidMiner 9.6.0 (RapidMiner lnc,Boston, USA). For WES, BWA (v0.7.12, Li H et al.), SAMtools (v1.9, Li Het al.) and Picard (v3.1.0, http://broadinstitute.github.io/picard/) were used to genome alignment, and muTect Software (Cibulskis K et al. 2013) was used for targeting Somatic SNV sites. For RNA-Seq, raw data quality was assessed with the FastQC (v0.11.9) and the adaptor was trimmed with Trim_Galore (version 0.6.6). Reads were mapped by using STAR software (v2.7.7a). The mapped reads were assembled into transcripts or genes by using StringTie software (v2.1.4). Valid sequencing data was mapped to the reference human genome (UCSC hg19) by Burrows-Wheeler Aligner (BWA, v0.7.12) software to get the original mapping results stored in BAM format. SAMtools (v1.9) and Picard (v3.1.0) were used to sort BAM files and do duplicate marking, local realignment, and base quality recalibration to generate final BAM file for computation of the sequence coverage and depth. Somatic variants were then called, utilizing VarScan v2.3.8, MuTect v1.1.7) and InVEX (http://www.broadinstitute.org/software/invex/). SCNA analysis was performed by following somatic copy-number variation (CNV) calling pipeline in GATK's (GATK v 4.1.2.0) Best Practice. The results of this pipeline, segment files of every 1,000, were put in GISTIC2 (v2.0). Mutational signatures were jointly inferred for tumors using the R package sigminer (v2.2.0) and for each sample was deconstructed using the deconstructSigs approach and its R package (deconstructSigs v1.8.0) with default parameters. For RNA-seq raw data quality was assessed with the FastQC (v0.11.9) and the adaptor was trimmed with Trim_Galore (v0.6.6) before any data filtering criteria was applied. Reads were

mapped onto the human reference genome (GRCh38.p13 assembly) by using STAR software (v2.7.7a). The mapped reads were assembled into transcripts or genes by using StringTie software (v2.1.4) and the genome annotation file (hg38_ucsc.annotated.gtf). Kinase activity scores were inferred from phosphorylation sites by employing PTM signature enrichment analysis (PTM-SEA) using the PTM signatures database (PTMsigDB) v1.9.0 (https://github.com/broadinstitute/ssGSEA2.0). GSEA was performed by the GSEA 4.0.3 software (http://software.broadinstitute.org/gsea/ index.jsp). To investigate the impact of different biological processes pathway enrichment on immune clusters, the "GSVA" R package (v1.42.0) was used to conduct GSVA enrichment analysis. All the analyses of clinical data were performed in R (v3.5.1) and GraphPad Prism 8 software. For functional experiments, each was repeated at least three times independently, and results were expressed as mean ± SEM. Statistical analysis was performed using GraphPad Prism 8 software.

For manuscripts utilizing custom algorithms or software that are central to the research but not yet described in published literature, software must be made available to editors and reviewers. We strongly encourage code deposition in a community repository (e.g. GitHub). See the Nature Portfolio guidelines for submitting code & software for further information.

Data

Policy information about availability of data

All manuscripts must include a data availability statement. This statement should provide the following information, where applicable:

- Accession codes, unique identifiers, or web links for publicly available datasets
- A description of any restrictions on data availability
- For clinical datasets or third party data, please ensure that the statement adheres to our policy

All the mass spectrometry proteome and phosphoproteome raw datasets have been deposited to the ProteomeXchange Consortium (dataset identifier: PXD043775) via the iProX partner repository (https://www.iprox.cn/) under Project ID: IPX0004596000. The raw WES and RNA data are available in the Genome Sequence Archive (GSA) under restricted access HRA004224 (https://ngdc.cncb.ac.cn/gsa-human/browse/HRA004224). The raw sequencing data are available under controlled access due to data privacy laws related to patient consent for data sharing and the data should be used for research purposes only. According to the guidelines of GSA-human, all non-profit researchers are allowed access to the data, and the Principle Investigator of any research group can apply for Controlled access of the data. The user can register and login to the GSA database website (https://ngdc.cncb.ac.cn/gsa-human/) and follow the guidance of "Request Data" to request the data step by step (https://ngdc.cncb.ac.cn/gsa-human/document/GSA-Human_Request_Guide_for_Users_us.pdf). The approximate response time for accession requests is about 2 weeks. The access authority can be obtained for Research Use Only. The user can also contact the corresponding author directly. Once access has been granted, the data will be available to download for 3 months. Human reference genome (GRCh38.p13 assembly) was downloaded from NCBI (https://www.ncbi.nlm.nih.gov/assembly/GCF_000001405.39/). TCGA BLCA data were downloaded from Xena (https://xenabrowser.net/). UROMOL cohort data were downloaded from the European Genome-phenome Archive (https://ega-archive.org/) under the accession code EGAS00001004693. The information of kinase-substrate relationships were available in PhosphoSite (https://www.phosphosite.org/homeAction.action), Phos-pho.ELM (http://phospho.elm.eu.org/dataset.html), and PhosphoPOINT (http://kinase.bioinformatics.tw/).The remaining data are available within the Article, Supplementary Information, and Source Data file. Source data are provided with this paper.

Human research participants

Policy information about studies involving human research participants and Sex and Gender in Research.

Reporting on sex and gender	A total of 190 participants (gender: 150 males and 40 females; age range: 35–92 years) were randomly recruited from patients who underwent surgical resection from January 2011 to December 2019.The sex ratio of bladder cancer patients was consistent with published papers in the bladder cancer field (PMID: 30096301; PMID: 24476821).
Population characteristics	The adjacent morphologically normal urothelial tissue (Normal), hyperplasia, urothelial proliferation of uncertain malignant potential (UPUMP), CIS, non-invasive low-grade papillary cancer (LGPC), non-invasive high-grade papillary cancer (HGPC), papilloma, invasive cancer without otherwise specified histology (NOS) or with variant histology (Variant) used in this study were obtained from the Zhongshan Hospital, Fudan University. A total of 190 patients (gender: 150 males and 40 females; age range: 35–92 years) were randomly recruited from patients who underwent surgical resection from January 2011 to December 2019.
Recruitment	A total of 190 participants (gender: 150 males and 40 females; age range: 35–92 years) were randomly recruited from patients who underwent surgical resection from January 2011 to December 2019.<br>There was no selection bias.
Ethics oversight	The study was approved by the Research Ethics Committees of Zhongshan Hospital (B2019-200R), and written, informed consent was provided by all patients.

Note that full information on the approval of the study protocol must also be provided in the manuscript.

Field-specific reporting

Please select the one below that is the best fit for your research. If you are not sure, read the appropriate sections before making your selection.

☒ Life sciences ☐ Behavioural & social sciences ☐ Ecological, evolutionary & environmental sciences

For a reference copy of the document with all sections, see nature.com/documents/nr-reporting-summary-flat.pdf

Life sciences study design

All studies must disclose on these points even when the disclosure is negative.

Sample size	The main purposes of this study were to investigate the molecular changes that occurred during the progression of urothelial bladder cancer and to explore the related effects of genomic aberrations on proteins and phosphoproteins during this process simultaneously. Based on the research objectives, the selection of samples for proteomic, phosphoproteomic, genomic, and transcriptomic studies needed to satisfy the following three principles: Firstly, all the samples needed to be conducted on proteomic profiling. Secondly, after ensuring proteomic profiling, the samples underwent phosphoproteomic profiling and whole-exome sequencing as much as possible. Finally, if there were any remaining samples, transcriptomic sequencing was conducted. Therefore, we performed a comprehensive proteomic, phosphoproteomic, genomic, and transcriptomic analysis to profile the proteogenomic patterns of samples dissected from urothelial bladder cancer of different stages and grades in 190 patients. Although the amount of tissue samples for studying urothelial bladder cancer (UC) progression was tiny, we still obtained 448 samples (190 cases) for proteomic profiling, 211 samples (139 cases) for phosphoproteomic profiling, 125 samples (125 cases) for whole-exome sequencing, and 67 samples (43 cases) for transcriptomic sequencing. No statistical method was used to predetermine sample size. The sample size of urothelial bladder cancer patients was based on published papers in the bladder field (PMID: 30096301; PMID: 24476821).Written informed consent was received from all patients included in this study.
Data exclusions	No data were excluded from the analyses.
Replication	All experiments were reliably reproduced and results are represented as mean ± SEM or mean ± SD as appropriate, which is indicated in figure legends. The replicated analysis of 293T cell lysates were used for the quality control of the mass spectrometer. 2 biological replicates for urothelial bladder cancer cell lines to obtain reliable results of cell proliferation and cell invasion.
Randomization	The UC samples for multi-omics processing were randomized, as investigators were blinded to clinical information.
Blinding	The investigators who measured protein expression, mRNA expression, and WES data were blinded to patient information. The investigators who performed IHC were blinded to clinical information of patients. For principal component analysis, consensus clustering analyses, all investigators were blinded to clinical information (including age, gender, tumor grade, tumor stage, pathological subtypes).

Reporting for specific materials, systems and methods

We require information from authors about some types of materials, experimental systems and methods used in many studies. Here, indicate whether each material, system or method listed is relevant to your study. If you are not sure if a list item applies to your research, read the appropriate section before selecting a response.

Materials & experimental systems

n/a	Involved in the study	
☐	☒	Antibodies
☐	☒	Eukaryotic cell lines
☒	☐	Palaeontology and archaeology
☐	☒	Animals and other organisms
☒	☐	Clinical data
☒	☐	Dual use research of concern

Methods

n/a	Involved in the study	
☒	☐	ChIP-seq
☒	☐	Flow cytometry
☒	☐	MRI-based neuroimaging

Antibodies

Antibodies used	Anti-ACOX1 (Proteintech, Catalog: 10957-1-AP, dilution 1:500),<br>Anti-RNASE2 (SAB, Catalog: SAB 42307, dilution 1:1000),<br>Anti-Flag antibody (Abcam, Catalog: ab205606, dilution 1: 1000),<br>Anti-Myc antibody (Abcam, Catalog: ab185656, dilution 1: 2000),<br>Anti- c-Jun (Proteintech, Catalog: 51151-1-AP, dilution 1:1000),<br>Anti- c-Fos (Proteintech, Catalog: 66590-1-Ig, dilution 1:1000),<br>Anti- histone H3 (Proteintech, Catalog: CL647-66863, dilution 1:1000),<br>Anti- Lamin (Proteintech, Catalog: 12987-1-AP, dilution 1: 5000),<br>Anti-RBPMS (Proteintech, Catalog: 15187-1-AP, dilution 1:1000),<br>Anti-rabbit IgG (Cell Signaling Technology, Catalog: 7074, dilution 1:1000).
Validation	Anti-ACOX1 (Proteintech, Catalog: 10957-1-AP, dilution 1:500) validated for immunohistochemistry by manufacturer [https://www.ptgcn.com/products/AOX-Antibody-10957-1-AP.htm],<br>Anti-RNASE2 (SAB, Catalog: SAB 42307, dilution 1:1000) validated for immunohistochemistry by manufacturer [https://www.sabbiotech.cn/g-15992-Non-secretory-ribonuclease-Polyclonal-Antibody-42307.html],<br>Anti-Flag antibody (Abcam, Catalog: ab205606, dilution 1: 1000) validated for western blotting by manufacturer [https://www.abcam.cn/products/primary-antibodies/ddddk-tag-binds-to-flag-tag-sequence-antibody-epr20018-251-ab205606.html],<br>Anti-Myc antibody (Abcam, Catalog: ab185656, dilution 1: 2000) validated for western blotting by manufacturer [https://www.abcam.cn/products/primary-antibodies/c-myc-phospho-s62-antibody-epr17924-ab185656.html],

	Anti- c-Jun (Proteintech, Catalog: #51151-1-AP, dilution 1:1000) validated for western blotting by manufacturer [https://www.ptgcn.com/products/JNK-Antibody-51151-1-AP.htm], Anti- c-Fos (Proteintech, Catalog: 66590-1-Ig, dilution 1:1000) validated for western blotting by manufacturer [https://www.ptgcn.com/products/FOS-Antibody-66590-1-Ig.htm], Anti- histone H3 (Proteintech Group, Catalog: CL647-66863, dilution 1:1000) validated for western blotting by manufacturer [https://www.ptgcn.com/products/Phospho-Histone-H3-Ser10-Antibody-CL647-66863.htm], Anti-Lamin (Proteintech, Catalog: 12987-1-AP, dilution 1: 5000) validated for western blotting by manufacturer [https://www.ptgcn.com/products/LMNB1-Antibody-12987-1-AP.htm], Anti-RBPMS (Proteintech, Catalog: 15187-1-AP, dilution 1:1000) validated for western blotting by manufacturer [https://www.ptgcn.com/products/RBPMS-Antibody-15187-1-AP.htm], Anti-rabbit IgG (Cell Signaling Technology, Catalog: 7074, dilution 1:1000) validated for western blotting by manufacturer [https://www.cellsignal.cn/products/secondary-antibodies/anti-rabbit-igg-hrp-linked-antibody/7074?site-search-type=Products&N=4294956287&Ntt=7074&fromPage=plp&_requestid=960541].

Eukaryotic cell lines

Policy information about cell lines and Sex and Gender in Research

Cell line source(s)	Human HEK293T (Cat# CRL-11268 from ATCC; RRID: CVCL_QW54), 5637 (Cat# HTB-9 from ATCC, RRID: CVCL_0126), and T24 (Cat# HTB-4 from ATCC; RRID: CVCL_0554), were obtained.
Authentication	All cell lines were routinely tested for mycoplasma contamination and authenticated by Short Tandem repeat (STR) profiling.
Mycoplasma contamination	All cell lines tested negative for mycoplasma contamination.
Commonly misidentified lines (See ICLAC register)	No commonly misidentified cell lines were used.

Animals and other research organisms

Policy information about studies involving animals; ARRIVE guidelines recommended for reporting animal research, and Sex and Gender in Research

Laboratory animals	Four-to-six-week-old Balb/C nude male mice were obtained from Shanghai SLAC Laboratory Animal Co., Ltd for in vivo xenografts. Mice were housed in pathogen-free, temperature-controlled environment, scheduled with 12-12 h light-dark cycles. The feeding conditions were specific pathogen free animal laboratory with 28 °C and 50% humidity 12/12, providing sufficient water and diet.
Wild animals	This study did not involve wild animals.
Reporting on sex	The mice used in the study were all male because bladder cancer cases occur more frequently in men, accounting for 80% of cases (PMID: 32972792).
Field-collected samples	The study did not involve field-collected samples.
Ethics oversight	For animal experiment, this study is under the guidelines of the animal care regulations of Fudan University, and was approved by Research Ethics Committee of department of experimental animal science, Fudan University.

Note that full information on the approval of the study protocol must also be provided in the manuscript.
